# Inappropriate implantable cardioverter defibrillator shocks induced by capacitive–resistive electric transfer therapy: the first documented case report of electromagnetic interference with electrogram evidence

**DOI:** 10.1093/ehjcr/ytag209

**Published:** 2026-03-14

**Authors:** Jacopo Costantino, Massimiliano Campoli, Barbara Romani, Lorenzo Maria Zuccaro, Daniele Porcelli

**Affiliations:** Department of Cardiology, Ospedale San Pietro Fatebenefratelli, Via Cassia 600, 00189 Rome, Italy; Department of Medical and Cardiovascular Sciences, Sapienza University of Rome, Viale del Policlinico 155, 00162 Rome, Italy; Department of Medicine, University of Padova, Via Giustiniani 2, 35128 Padua, Italy; Department of Cardiology, Ospedale San Pietro Fatebenefratelli, Via Cassia 600, 00189 Rome, Italy; Department of Cardiology, Ospedale San Pietro Fatebenefratelli, Via Cassia 600, 00189 Rome, Italy; Department of Cardiology, Ospedale San Pietro Fatebenefratelli, Via Cassia 600, 00189 Rome, Italy; Department of Cardiology, Ospedale San Pietro Fatebenefratelli, Via Cassia 600, 00189 Rome, Italy

**Keywords:** TECAR therapy, ICD, Electromagnetic interference, Inappropriate shock, Case Report

## Abstract

**Background:**

Capacitive–resistive electric transfer (TECAR) therapy is a form of endogenous diathermy delivering radiofrequency current and is increasingly used for musculoskeletal rehabilitation. However, data regarding its safety in patients with cardiac implantable electronic devices (CIEDs) are limited.

**Case summary:**

A 54-year-old man with a dual-chamber implantable cardioverter defibrillator (ICD) underwent lumbar TECAR therapy for muscular contracture. During the session, he experienced two inappropriate ICD shocks while remaining conscious and without clinical sequelae. Device interrogation revealed high-frequency, non-physiological signals simultaneously recorded on atrial and ventricular intracardiac electrograms (EGMs), which were interpreted by the ICD as ventricular fibrillation and resulted in shock delivery.

**Discussion:**

This report represents the first documented case demonstrating EGM evidence of electromagnetic interference between TECAR therapy and an ICD. The findings highlight a relevant safety concern and suggest that TECAR therapy should be avoided in ICD carriers and, more broadly, used with extreme caution in patients with other CIEDs, only after careful risk–benefit assessment and discussion with an electrophysiology specialist.

Learning points

- Patients with cardiac implantable electronic devices should always disclose their device status, and physiotherapists must verify it before any electrotherapeutic procedure.
- Capacitive–resistive electric transfer therapy may generate clinically relevant electromagnetic interference and should be avoided in implantable cardioverter defibrillator carriers due to the risk of inappropriate shocks.
- Close communication between electrophysiologists and rehabilitation professionals is essential to prevent unintended exposure of cardiac implantable electronic device patients to contraindicated energy-based therapies.

## Introduction

Capacitive–resistive electric transfer (TECAR) therapy is a diathermy technique that applies radiofrequency currents (300–500 kHz) through body tissues using an active and a return electrode.^1^ It is increasingly adopted in musculoskeletal rehabilitation to promote local heating, pain relief, and tissue recovery.^2^ Although TECAR operates at lower frequencies than traditional diathermy, it still generates electromagnetic fields capable of interacting with cardiac implantable electronic devices, and its safety in implantable cardioverter defibrillator (ICD) carriers has never been evaluated. We report the first case of inappropriate ICD shocks induced by TECAR therapy, supported by intracardiac electrograms (EGMs) illustrating how an ICD interprets radiofrequency interference from this therapeutic modality.

## Summary figure

**Figure:**
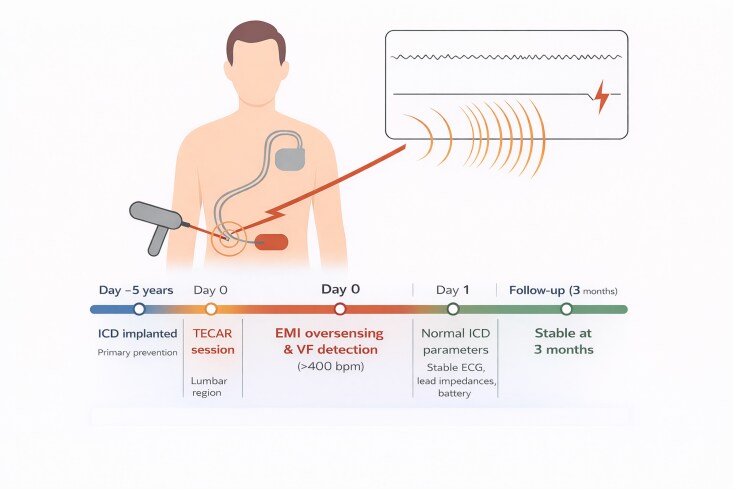


## Case presentation

**Figure 1:**
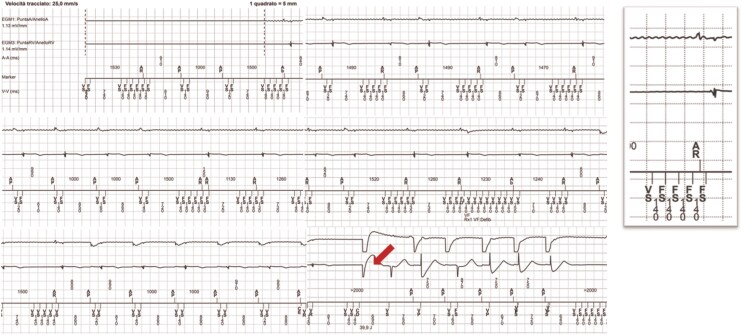
Stored intracardiac electrogram recorded during the capacitive–resistive electric transfer therapy session. The tracing shows the first inappropriate shock (arrow) delivered by the device. The inset on the right displays a magnified view of the high-frequency electromagnetic noise simultaneously recorded on atrial and ventricular channels (A tip–A ring; RV tip–RV ring). Detection markers displayed on the tracing include: AP, atrial paced event; AR, atrial sensed refractory event; CE, charge end; CD, capacitor discharge (shock delivery); FS, ventricular fibrillation sense; VS, ventricular sensed event; VR, ventricular sensed refractory event. Sweep speed: 25 mm/s; 1 large square & 5 mm.

A 54-year-old man with non-ischaemic dilated cardiomyopathy (left ventricular ejection fraction 30%) had received a dual-chamber Medtronic Crome DR (model DDPC3D4) ICD 5 years earlier for primary prevention of sudden cardiac death. Device performance and lead integrity had remained stable at all prior follow-ups. In July 2025, the patient underwent TECAR therapy for a lumbar muscular contracture. The session was performed in the resistive mode, with the active electrode applied over the lower back and the return plate positioned on the anterior abdominal wall. The patient did not inform the physiotherapist of his ICD implantation. A few minutes after the session began, he experienced two abrupt, painful ICD shocks in rapid succession, without loss of consciousness or haemodynamic compromise. Device interrogation revealed one ventricular fibrillation (VF) episode treated with two 40 J shocks (*Figure 1*).

Stored intracardiac EGMs provided clear evidence of electromagnetic interference (EMI).

- On the atrial channel (A tip–A ring), low-amplitude oscillatory activity consisting of 8–10 fine deflections per paced atrial interval was observed.
- On the ventricular channel (RV tip–RV ring), bursts of small, high-frequency potentials (5–10 oscillations) were superimposed on otherwise normal ventricular EGMs and annotated as ventricular sensed and fibrillatory sensed events, producing an apparent ventricular rate >400 b.p.m. within the programmed VF zone.

After the shocks, normal sensing resumed immediately, with no recurrence. Lead impedances, pacing thresholds, and battery parameters remained unchanged. At the time of the event, the ICD was programmed in the DDD mode with a VF detection threshold of 290 ms and delivered two 40 J shocks without antitachycardia pacing. On admission, the patient was alert, asymptomatic, and haemodynamically stable, with normal vital signs and an unremarkable cardiovascular examination. At 3-month follow-up, he remained clinically stable, with unchanged device parameters and no further inappropriate therapies. He was educated about contraindicated procedures and advised to inform all healthcare providers of his ICD; the physiotherapy centre was also notified.

## Discussion

This case represents the first published documentation of inappropriate ICD shocks induced by TECAR therapy, a modern form of capacitive–resistive diathermy. The stored EGMs provide direct electrophysiological evidence of how an ICD senses EMI during this treatment.

Capacitive–resistive electric transfer devices deliver alternating radiofrequency current between an active and a passive electrode, producing deep tissue heating by displacement currents.^2^ Therapy can be delivered in the capacitive mode, which preferentially targets superficial, water-rich tissues through insulated electrodes, or in the resistive mode, which employs non-insulated electrodes to allow deeper current penetration into higher-resistance tissues such as muscles and joints.^1,2^

When applied to regions where the electric field intersects the thoraco-abdominal pathway, as in this case, the generated electromagnetic energy may couple with ICD leads, inducing high-frequency artefacts sensed as ventricular activity. The EGM morphology—simultaneous, uniform, high-frequency oscillations on both atrial and ventricular channels without true QRS structure—is pathognomonic for EMI rather than intrinsic arrhythmia, lead noise, or myopotentials.^3^

Modern ICDs incorporate multiple discrimination algorithms to reduce inappropriate therapies by distinguishing ventricular tachyarrhythmias from supraventricular tachycardias (SVT) and oversensing.^4^ In Medtronic Crome devices, SVT discrimination relies on PR Logic™ (atrioventricular relationship analysis) together with onset and stability criteria. Wavelet is a morphology-based discriminator that compares the ventricular EGM during tachycardia with a stored template; a high degree of similarity supports an SVT mechanism and may inhibit VT detection within programmable limits. To mitigate therapies triggered by non-physiological signals, these devices also include ventricular oversensing discriminators, such as RV lead noise discrimination, which compare near-field RV sensing with a far-field EGM during early detection phases. However, discrimination algorithms are primarily intended for ventricular tachycardia zones, whereas in the VF detection zone, ICDs are designed to prioritize sensitivity over specificity. Once VF detection criteria are fulfilled, SVT and morphology-based discriminators, including Wavelet, are not applied to avoid withholding life-saving therapy. In the present case, TECAR-related EMI generated sustained high-frequency, non-physiological signals simultaneously recorded on atrial and ventricular channels, fulfilling VF detection criteria. Under these conditions, noise discrimination algorithms are inherently limited, as intense EMI may be classified as VF to preserve device sensitivity, leading to shock delivery.

The morphology and periodicity of the intracardiac EGMs may reflect the energy delivery pattern of the TECAR generator, which operates at carrier frequencies of 300–500 kHz with pulsed emission cycles. In our patient, repetitive trains of uniform high-frequency deflections intermittently visible on both atrial and ventricular channels were more consistent with pulsed energy delivery than with continuous electromagnetic noise. Although direct synchronization was not recorded, this temporal pattern supports a plausible mechanistic link between TECAR energy delivery and ICD oversensing.

While EMI from shortwave or microwave diathermy is well documented, TECAR therapy has never been formally studied in the context of cardiac device safety.^5^ No clinical or bench-top investigations have assessed interference thresholds or coupling effects on CIEDs, and available data are largely outdated. Nevertheless, TECAR operating frequencies and current delivery systems are fundamentally comparable to those of traditional diathermy.

Current manufacturer recommendations list all forms of diathermy as contraindicated in patients with CIEDs. This case demonstrates that TECAR should be regarded within the same safety framework, as it can induce ICD oversensing even when applied to anatomical sites distant from the pulse generator. The increasing diffusion of TECAR in rehabilitation medicine highlights the need for awareness, as patients and physiotherapists must recognize that TECAR, like all diathermy, can produce hazardous EMI in ICD carriers. This case underscores the importance of interdisciplinary communication between electrophysiologists and rehabilitation professionals to prevent inadvertent exposure of CIED patients to contraindicated energy-based therapies.

## Conclusion

Capacitive–resistive electric transfer therapy is an electrotherapeutic modality that emits radiofrequency energy capable of interacting with implantable cardiac devices. This first reported case of inappropriate ICD shocks caused by TECAR interference demonstrates, through stored electrograms, that even short exposures can trigger full ICD therapy delivery. Until dedicated safety studies are available, TECAR should be avoided in ICD carriers and approached with extreme caution in patients with other CIEDs, only after careful risk–benefit assessment and discussion with an electrophysiology specialist.

## Data Availability

Data available on request—the data underlying this article will be shared on reasonable request to the corresponding author.

## Lead author biography

Jacopo Costantino is a young cardiologist specializing in cardiac electrophysiology and cardiomyopathies. He is currently a Research Fellow at the Department of Cardiology, Sapienza University of Rome, and a PhD candidate at the University of Padua. He works with the Electrophysiology Laboratory of San Pietro Fatebenefratelli Hospital in Rome.

## Notes

**Consent:** The authors confirm that written informed consent for the publication of this case report and any accompanying images was obtained from the patient in accordance with COPE.
